# Photoluminescence and Boosting Electron–Phonon Coupling in CdS Nanowires with Variable Sn(IV) Dopant Concentration

**DOI:** 10.1186/s11671-021-03485-3

**Published:** 2021-01-29

**Authors:** Yuehua Peng, Yuan Luo, Weichang Zhou, Xuying Zhong, Yanling Yin, Dongsheng Tang, Bingsuo Zou

**Affiliations:** 1 School of Physics and Electronics, Key Laboratory of Low-Dimensional Quantum Structures and Quantum Control of Ministry of Education, Key Laboratory for Matter Microstructure and Function of Hunan Province, Synergetic Innovation Center for Quantum Effects and Application, Hunan Normal University, Changsha, 410081 People’s Republic of China; 2 School of Physical Science and Technology, MOE Key Laboratory of New Processing Technology for Non-Ferrous Metals and Materials, Guangxi Key Laboratory of Processing for Non-Ferrous Metals and Featured Materials, Guangxi University, Nanning, 530004 People’s Republic of China

**Keywords:** CdS nanowires, Doping, Photoluminescence, Electron–phonon interaction

## Abstract

High-quality Sn(IV)-doped CdS nanowires were synthesized by a thermal evaporation route. Both XRD and Raman scattering spectrum confirmed the doping effect. The room temperature photoluminescence (PL) demonstrated that both near bandgap emission and discrete trapped-state emission appeared simultaneously and significantly, which were attributed to the strong exciton trapping by impurities and electron–phonon coupling during the light transportation. The PL intensity ratio of near bandgap emission to trapped-state emission could be tune via doped Sn(IV) concentration in the CdS nanowires. It is interesting that the trapped-state emission shows well separated peaks with the assistance of 1LO, 2LO, 4LO phonons, demonstrating the boosting electron–phonon coupling in these doped CdS nanowires. The influence of Sn(IV) dopant is further revealed by PL lifetime decay profile. The optical micro-cavity also plays an important role on this emission process. Our results will be helpful to the understanding of doping modulated carrier interaction, trapping and recombination in one-dimensional (1D) nanostructures.

## Introduction

Recently, one-dimensional (1D) nanowires were attractive because they can function as both building block devices and integrated nanosystems [1–4]. Specially, 1D single-crystalline wide-bandgap II–VI semiconductors, such as CdS with a direct band gap of 2.6 eV at room temperature, were studied widely due to their excellent photonics, electronics and optoelectronics properties. There are lots of reports on the CdS nanowire/nanobelt lasers, optical waveguides, photodetectors, field effect transistors and logic devices [5–8]. Many intrinsic carrier interactions, such as: electron–hole plasma, electron–phonon coupling, exciton–phonon scattering, exciton–exciton interaction and excitonic polariton, take effect in a new way in the nanometer-scale [9–13]. The strength of related interaction depended strongly on the size of nanostructures, therefore influencing the optical/optoelectronic properties greatly [14]. Such as, the quantum confinement effect and electron–phonon coupling would become especially important when introducing the localized states.

Bandgap engineering via adjust size or composition is usually adopted to realize the variable lasing and luminescence wavelength/color. As a polar semiconductor with the electron-LO phonon coupling constant of 0.65, exciton energy modification in CdS nanostructures is expected due to the strong Fröhlich interaction and deformation potential [15]. Such as, Zou et al. [16] reported the bipolaronic excitons stimulated emission in single CdS nanowires at room temperature. Lieber et al. [17] reported an exciton–exciton interaction for lasing in CdS nanowires up to 75 K, while an exciton–phonon process at higher temperature. In addition, electron–hole plasma (EHP) and Fabry–Perot (F-P) optical resonant processes could also responsible for stimulated emission of aligned CdS nanowires, although the tunable wavelength range is small and the EHP often damages the nanowires [18]. These examples demonstrated that tuning of Fröhlich electron–phonon coupling along the 1D axial light propagation was a plausible way to realize variable emission or lasing wavelength. Recently, branched CdS nanowires have been grown via Sn nanowire-template route under thermal annealing and show interesting optical waveguide properties [19].

Here in this paper, we report on the synthesis of Sn–CdS nanowires by using SnO_2_ as catalyst and dopant, and their anomalous PL and electron–phonon coupling properties. Both the near bandgap emission and in-gap emissions appear simultaneously in the PL of as-synthesized nanowires. The latter even show cavity-related waveguide modes. The reasons for these phenomena originate from Sn(IV) doping into CdS nanowires to produce many trapping centers. The coupling of electron with phonon leads to anomalous emission enhancement with waveguide and color tuning in a very large range.

## Methods

### Synthesis of Sn-Doped CdS Nanowires

Thermal evaporation was used to synthesize the Sn-doped CdS nanowires. Commercial CdS and SnO_2_ powder with 1:1 weight ratio were fully mixed by grinding for 30 min and then used as source material, which was loaded on an alumina ceramic boat and placed at the center of the quartz tube. Changing the weight ratios of CdS to SnO_2_ powder was used to grow CdS nanowires with different Sn dopant concentration. Clean Si wafers with no catalyst were placed at the downstream zone to collect product. A mixture carrier gas of Ar(95%)/H_2_(5%) was introduced into the quartz tube with a constant flow rate of 10 SCCM (standard cubic centimeters per minute) to remove O_2_ inside before heating. H_2_ might produce a reductive atmosphere and can prevent oxidation and improve yield of CdS nanowires. The furnace was rapidly heated to 1000 °C within 10 min and maintained at that temperature for 60 min without changing any conditions before cooling down to room temperature naturally. The synthesized products were found on the surface of Si substrate and inner wall of quartz tube in a zone 5 cm away from the source material, where the local growth temperature was in the range of 400–450 °C.

### Structure and Optical Properties Characterization

The structure, morphology and composition of as-obtained products were characterized with powder X-ray diffraction (XRD, Bruker D8 Advance), scanning electron microscope (SEM, JSM-6700F), energy-dispersive spectroscope (EDS). Raman scattering spectrum was performed in a confocal microscope (LABRAM-010) using He–Ne laser (632.8 nm) as the excitation light source, which was focused into a spot with diameter of 3 μm on the samples. Optical waveguide and PL were carried out using commercial scanning near-field optical microscopy (SNOM, Alpha 300, WITec). SNOM has the ability to achieve a high spatial resolution optical image of nanostructures and simultaneously measure its PL spectrum. With this equipment, a focused Ar^+^ laser (488 nm) beam illuminated on the single nanowire that was pre-deposited on the quartz substrate. A chromatic color CCD through an objective lens was used for collecting the optical image. PL spectra from a whole individual Sn-doped CdS nanowire were directly collected and coupled into the fluorescence spectrometer. In all optical experiment, the excitation signal illuminated perpendicularly onto the sample surface.

## Results and Discussion

**Fig. 1:**
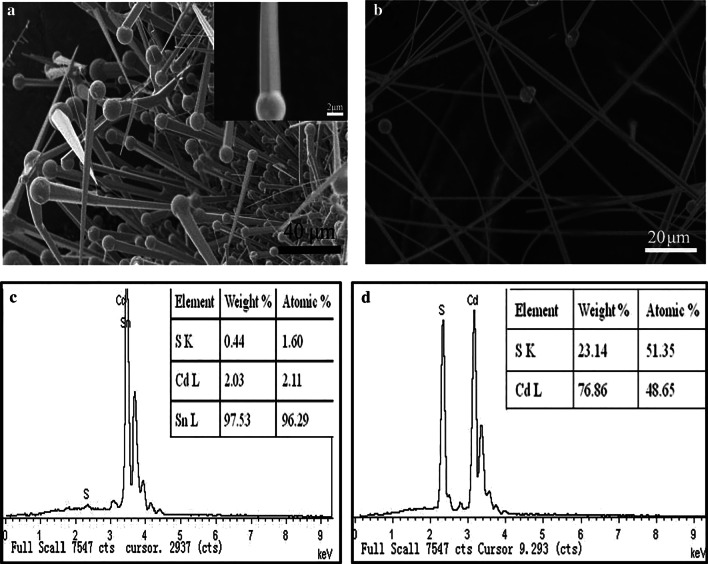
**a**, **b** SEM of Sn-doped CdS nanowires formed at early stage and after 60 min growth. Inset of **a** is amplified magnification of a representative nanowire. **c**, **d** EDS of the tipped ball and backbone nanowire, respectively

Figure 1a, b shows the morphology images of nanowires formed at early stage and 60 min growth time, respectively. The nanowires at early stage usually contain a big ball at the tip and a long wire connecting with the ball. The nanowire diameter ranges from 200 nm to 2 μm and length up to hundreds of micrometers. EDS analysis indicates the ball is Sn (Fig. 1c), while the wire is CdS dominantly (Fig. 1d). It is surprising that the Cd:S is always less to 1. The shake-up peak above the Cd element indicates the possible Sn distribution in the wires, although cannot be distinguished via EDS directly. It is known that SnO_2_ can decompose into Sn or Sn(IV) and O_2_, while CdS powder forms Cd, S and CdS vapor at high temperature. The as-formed Sn after decomposition may exist in the form of gas or small liquid droplets (mp 232 °C, bp 2602 °C). These gas or droplets are then transported to low temperature zone by carrier gas and deposit as a liquid layer, which react with arrived CdS vapor and result in Sn–CdS liquid alloy layer for initiation-doped CdS nanowires growth. Therefore, the nanowires follow vapor–liquid–solid (VLS) growth process [20], where the Sn catalyst particles or droplets are responsible for the growth of CdS nanowires as long as the alloy layer keep collecting the Sn and CdS vapor. The nanowires have tadpole-like morphology at early stage, and the surface is smooth. Liu et al. [21] synthesize branched CdS nanostructures by using such tiny Sn as catalyst and second nucleation site. In our experiment, Sn diffused and doped into the lattice of CdS nanowires and had not yet reach the super-saturation to precipitate as second nucleation site. So the as-prepared products possess straight morphology instead of branch junction.

**Fig. 2:**
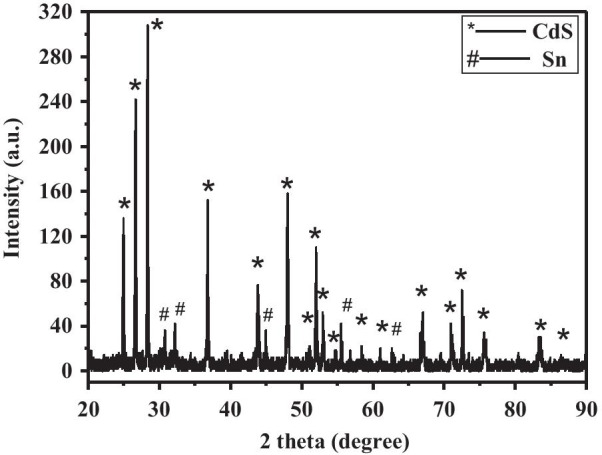
X-ray diffraction patterns of Sn-doped CdS nanowires

The X-ray diffraction pattern of as-prepared nanowires is shown in Fig. 2. The crystallographic phase is in good agreement with wurtzite hexagonal CdS (JCPDS card: 41-1049) with lattice constants of *a* = 4.141 Å and *c* = 6.720 Å. Hence, the as-prepared nanowires can be designated to wurtzite CdS. In addition, the diffraction peaks of Sn (JCPDS card: 4-673 for tetragonal with lattice constants of *a* = 5.831 Å and *c* = 3.182 Å) are clearly observed, indicating that a large amount of Sn exist on the head of CdS nanowires. These strong and sharp diffraction peaks show high crystal quality of as-prepared nanowires.

**Fig. 3:**
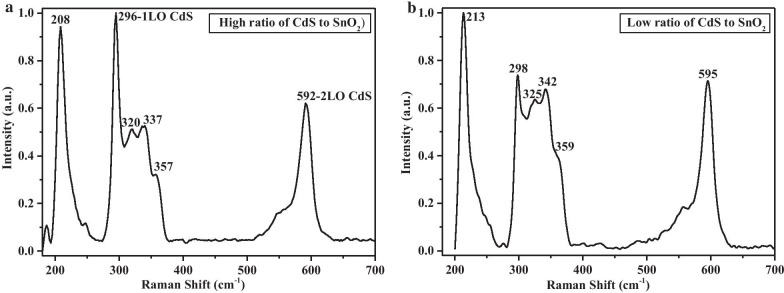
**a**, **b** Micro-Raman scattering spectra of single Sn-doped CdS nanowire synthesized with high and low ratio of CdS to SnO_2_, by using He–Ne laser (632.8 nm) as excitation light source

Figure 3a, b shows the micro-Raman scattering spectra of single nanowires with different dopant concentration at room temperature. The two strong peaks at 296 and 592 cm^−1^ are attributed to the 1LO and 2LO of CdS, respectively [22]. The polarization in the *Z* axis strongly couples with the electron or exciton. The strength of electron-LO phonon coupling in 1D semiconductor structures can be greatly strengthened due to the phonon confinement in transverse directions and the convenient transportation of elementary excitation (exciton and photon) in longitudinal direction. The strength of electron–phonon coupling in semiconductors can be assessed by the intensity ratio of overtone phonon to fundamental phonon ($I_{{{\text{2LO}}}} /I_{{{\text{1LO}}}}$) [23]. In our result, the large intensity ratio of $I_{{{\text{2LO}}}} /I_{{{\text{1LO}}}}$ (1.67) reflects a stronger electron-LO phonon coupling in these Sn–CdS nanowires than the 1D pure CdS nanowires with $I_{{{\text{2LO}}}} /I_{{{\text{1LO}}}}$ of 1.52. Four others phonon modes (208, 320, 337, 357 cm^−1^) were observed in the Raman scattering spectrum, which are not the intrinsic phonon modes of CdS. After careful examination, these modes were designated to $E_{{\text{g}}}$, $A_{{{\text{1g}}}}$, $A_{{2{\text{u}}({\text{TO}})}}$, $A_{{2{\text{u}}({\text{LO}})}}$ impurity vibration modes of doped Sn(IV)S_2_, respectively [24, 25]. Very interestingly, we can even observe the IR active modes ($A_{{2{\text{u}}({\text{TO}})}}$, $A_{{2{\text{u}}({\text{LO}})}}$) of SnS_2_ in the Raman spectrum, indicating a significant relaxation of transition rule under the electronic assistance, i.e., carrier trapping. As the crystals are uniaxial and when the phonon propagation direction is not along the principle axis of the crystal, some infrared and Raman modes both may be active simultaneously due to electron–phonon or exciton–phonon interaction [26]. The increased crystal deformation and surrounding structural fluctuation due to Sn dopant in the CdS nanowires contribute in part to the weakly active of infrared modes [27]. $A_{{2{\text{u}}}}$ is acoustic mode, whose occurrence implies the prominent interaction of electron with local acoustic phonon and therein contributes partially to the abnormal emission properties in Sn–CdS nanowires. The observed impurity vibration modes further suggest the successfully doping of Sn(IV) with definite amount into the nanowires, which modified the optical properties greatly. We executed X-ray photoelectron spectroscopy (XPS) measurement on these Sn–CdS nanowires. However, it cannot distinguish that the detected Sn content was from the tipped ball or the backbone nanowire due to the low spatial resolution of XPS. In addition, the Sn content is too low to detect by using energy-dispersive spectroscope (EDS). Here, we determined qualitatively the higher or lower Sn concentration in the backbone nanowire by using Raman spectra. As shown in the Raman spectra, the intensity ratio of CdS mode to SnS_2_ mode reduces with increased ratio of SnO_2_ to CdS in the source powder, indicating the highly doped Sn concentration qualitatively.

**Fig. 4:**
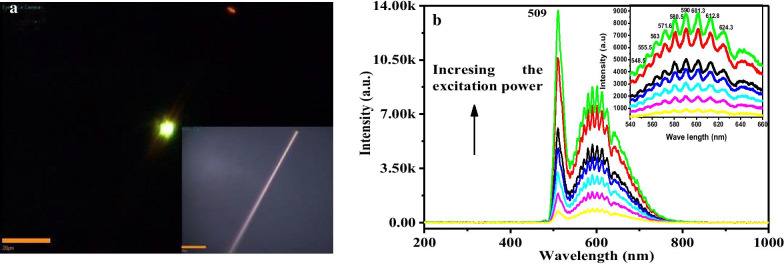
**a** Far-field emission image of single Sn-doped CdS nanowire. Inset is the bright-field optical image of corresponding single nanowire. Scale bars are 20 μm. **b** Far-field PL spectra under increasing excitation power. Inset is the local scale-up of impurities emission bands

PL measurement is very powerful for determination the structure defect and impurity in semiconductor nanostructures. For weak Fröhlich electron–phonon coupling system, the PL is modified by introducing dopant, which will affect the formation potential and photon–phonon coupling coefficient. The emission band can show multi-phonon replica when the dopant locates at deep level. The exciton emission band even collapses into broad band or satellite bands of deep trap state in strong coupling system. We measured the optical waveguide and PL spectrum of single straight Sn–CdS nanowire (dopant concentration < 0.01%) to explore the doping effect and electron–phonon coupling according the intensity ratio of near bandgap emission to trapped state emission. The in situ PL of single Sn–CdS nanowire was so strong that it could be imaged easily with a color CCD camera and was visible to naked eye (Fig. 4a). The bright-field optical image of corresponding Sn–CdS nanowire is shown in the inset of Fig. 4a. A portion of the emission can propagate along the axis and emit at the end of nanowire even under low excitation power, showing the excellent optical waveguide property of doped CdS nanowire. We can observe that the in situ emission color is yellow-green, while the emission color at the end is red after long distance transportation (Fig. 4a). Pan et al. reported the emission color changes with different distances in Se densely doped CdS 1D nanostructures and attributed it to band-tail shift due to composition and crystallization degree changes [28]. In fact, the effect of electron–phonon coupling enhancement cannot be ignored. In the present Sn sparsely doped CdS nanowires, the reason for emission color change originates from trapped excitons by impurities and enhanced e-p coupling during light transportation along *c* axis. Figure 4b is far-field excitation power-dependent PL. The emission at 509 nm is attributed to near bandgap emission, while the other broad emission bands at lower energy are attributed to trapped state emission. The intensities of all emission bands increase rapidly with excitation power, while the near bandgap emission increases only slightly faster than the trapped state emission. This phenomenon is different from the traditional deep trap state, where the near bandgap emission intensity increased greatly under increasing excitation power. The energy spans between adjacent trapped state bands are 229.7, 239.8, 267.3, 268.3, 277, 318.6, 312.1, 300.6 cm^−1^, which increase slightly to the longer wavenumber and approach to the energy of LO phonon (296 cm^−1^). This multi-phonon profile indicates a nonlinear e-p coupling of trapped state [29]. The trapped excitons still keep their coherence during the light transportation and scatter with doped tin ions to emission coherently. Thus, the slightly deviation of energy spans from LO phonon energy may due to the increasing light passing distance, indicating the incomplete localization of deep-trapped states. These excitons prefer to align along a line in the 2D confinement nanostructures and couple to form new exciton aggregation. Clearly, aggregation itself represents a nonlinear correlation between excitons and leads to giant oscillator strength, yielding nonlinear optical responses [30, 31]. For imperfect nanomaterial with plenty of impurities or defects, the impurities or defects-induced crystal deformation can remarkably modify the electron–phonon coupling and always induces *n*LO phonon-assisted emission, in addition to the near band gap emission [32–34]. This multi-phonon emission profile is similar to the high-order stimulated Raman processes in a silica fiber and their difference lies in the real state contribution for the emission band. It is reported that the number (*n*) of multiple phonon scattering processes is in proportion to the polaron coupling coefficient *α*, that is, the maximum frequency shift $n\omega_{{{\text{LO}}}}$ is proportional to the deformation energy (0.5 $\alpha h\omega_{{{\text{LO}}}}$) [35]. Therefore, the present PL spectra indicate a very large deformation potential and strong electron–phonon coupling in Sn–CdS nanowires.

**Fig. 5:**
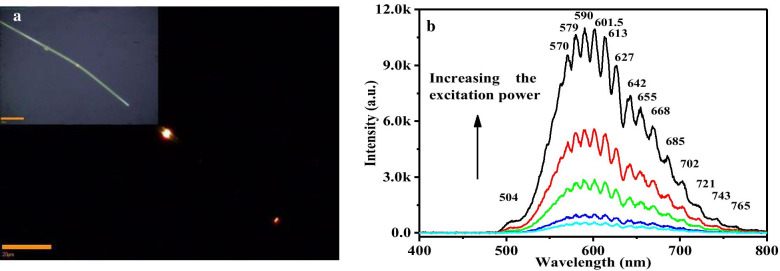
**a** Far-field emission image of single Sn(IV) heavy slightly doped CdS nanowire. Inset is optical morphology image. Scale bars are 20 μm. **b** The corresponding far-field PL spectra under increasing excitation power

The concentration of doped Sn(IV) in CdS nanowires increased with the ratio of SnO_2_ to CdS in precursor, yielding similar PL spectra with much weaker near bandgap emission while stronger impurity emission, as shown in Fig. 5. This represents enhanced e-p coupling in this system. Different from Fig. 4b, the intensity ratios of near bandgap to trapped state emission decrease to 0.05–0.10 with variable excitation power. The trapped state emission intensity goes up faster than the near bandgap emission under increasing excitation power, demonstrating the stronger scattering relatively by impurity and phonon. The energy spans between adjacent impurities bands are 272.7, 325, 324, 311.9, 364.3, 372.6, 309.1, 297.1, 371.5, 375.4, 410.7, 387.1 cm^−1^, which approach to the LO phonon energy (296 cm^−1^). This larger deviation reflected the step-by-step excitation in the active light waveguide and demonstrated more Sn ions doping into the nanowires to produce stronger electron–phonon coupling.

**Fig. 6:**
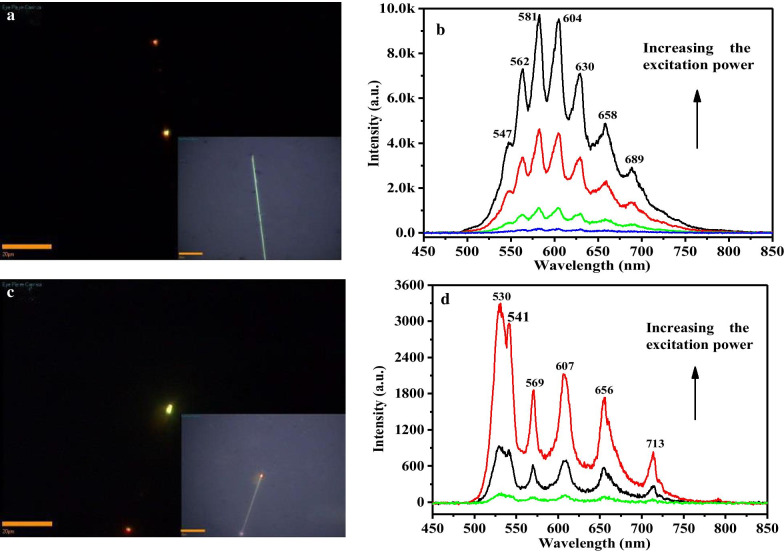
**a**, **c** Far-field emission and optical (inset) images of CdS nanowires with higher dopant concentration. Scale bars is 20 μm; **b**, **d** The corresponding far-field PL spectra

There is another interesting PL spectrum of Sn-doped CdS nanowires (Fig. 6). In the nanowires with higher doping concentration, the near bandgap emission is very weak, while the trapped state emission plays a dominant role. The energy spans between adjacent impurities emission bands are 488, 581.9, 655.4, 683.3, 683.8 cm^−1^, which increase slightly and close to 2LO phonon energy (592 cm^−1^). The 2LO phonon-assisted emission intensity shows significant increase with excitation power. The appearance of 2LO phonon-assisted emission suggests the strong electron-2LO phonon coupling, consistent well with the Raman scattering spectrum that showing strong 2LO Raman mode (Fig. 3). This interesting phenomenon cannot clearly be understood with usual concepts. It is reported that the longitudinal optical mode couples with electron within one picosecond [23]. If Sn-Sn pairs form in high doping situation, the electron-1LO phonon coupling may lead to a bound pairs like bipolaronic excitons at the bound location. Such bipolaronic excitons (electron-2LO phonon) states may emit light coherently, which often occur at the trapped center with long lifetime, while the 1LO-assisted emission band cannot be well resolved after a long transportation length and a short-time relaxation. Therefore, the 2LO-assisted emission may dominate in the higher doping concentration nanowires. More doping caused even more anomalous phenomenon. There may initiate structural dislocation and possible formation of cubic phase. Figure 6d shows a similar PL spectra of doped nanowire with further increasing Sn(IV) concentration. The emission bands at 530 nm and 541 nm may attribute to near-bandgap emission of wurtzite and zinc-blende phase CdS, respectively [36]. The energy difference $\Delta E^{{{\text{WZ}} - {\text{ZB}}}}$ is 0.048 eV in the present case, agreement with the fact that the bandgap energy difference of WZ and ZB CdS is less than 0.1 eV [37, 38]. The more important thing is the energy span of adjacent trapped state emission bands that showing values of 1100.2, 1230.6, 1218.7 cm^−1^, which are close to 4LO phonon energy (1184 cm^−1^). This overtone effect suggests higher-order electron–phonon coupling in the Sn–CdS nanowires.

**Fig. 7:**
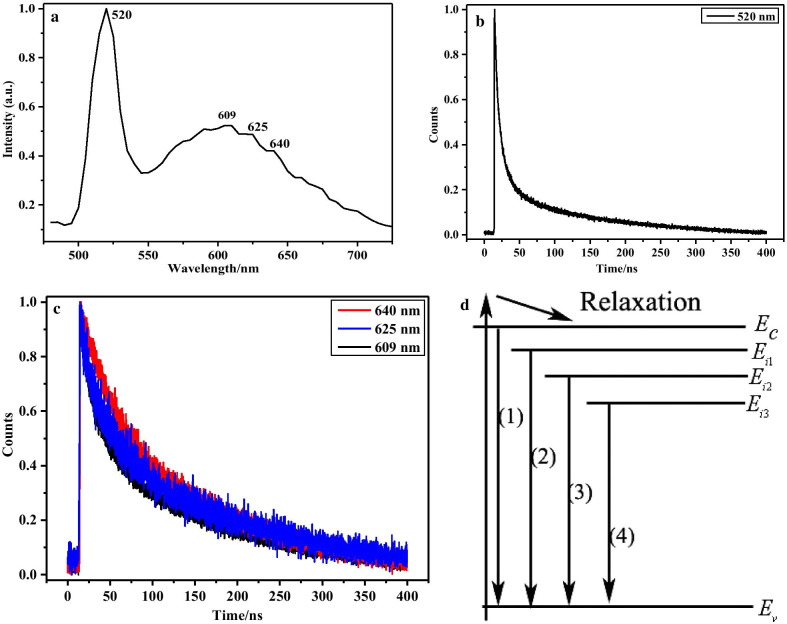
**a** PL of Sn–CdS nanowires under excitation of pulse laser with wavelength of 400 nm. **b**, **c** The corresponding PL lifetime decay profiles of 520 nm and 609, 625, 640 nm. **d** The schematic diagram for the carrier with different lifetime decay processes

To investigate the different emission mechanism, we executed PL decay measurement on the near bandgap emission and trapped state emission under excitation of ps dye N_2_ pulse laser at 400 nm (Fig. 7). The near bandgap emission locates at 520 nm, while the deep-trapped emission ranges from 550 to 750 nm and centers at 609 nm (Fig. 7a), consistent with the PL of single nanowire investigated by SNOM. The lifetime decay profiles of 520 nm and 609 nm, 625 nm, 640 nm are shown in Fig. 7b, c. The decay time of 520 nm and 609 nm were fitted with multi-exponential function, showing time constants of 0.3227 ns (22.90%), 4.2585 ns (25.86%), 26.4584 ns (35.31%), 289.1292 ns (15.92%) and 0.1309 ns (60.41%), 0.6641 ns (8.39%), 24.8286 ns (20.86%), 194.1492 ns (10.35%), respectively. The PL lifetime of 640 nm, 625 nm and 609 nm bands exhibited the almost same profiles and lifetime parameters. Such results are in contrast with the previous reported [18]. The energy relaxation process of pure CdS nanowire under different excitation power only undergoes electron–phonon and exciton–exciton interaction. For the Sn-doped CdS nanowires, other interaction like deep trapped state and cavity effect should participate in the relaxation process. A schematic diagram of decay processes is given in Fig. 7d. The charged carriers in the trap and exciton coexist and relax to conduction band edge from the higher extended band after excitation and lots of them are trapped by impurity states to within the band-gap. The interactions between carriers from these different impurity levels or paired state levels are also involve in the recombination of exciton and emit at the active optical cavity, corresponding to the observed different decay lifetimes. There are partial carriers to be scattered out from one deep-trapped level and are trapped again by the low trap level (the (1), (2), (3), (4) recombination process in Fig. 7d). For bandgap emission, the fast time constant such as 0.3227 ns should correspond to the direct recombination of carriers, while the other time constants may correspond to the radiative recombination of bound excitons (4.2585 ns) and shallow-trapped carriers between impurity energy band and valence band (tens and hundreds ns). For the trapped state emission, the much shorter lifetime of < 1 ns and others components reflected the rich carrier-carrier and carrier-phonon interactions, demonstrating the typical characteristics of trapped state.

## Conclusions

In summary, we synthesized the high-quality Sn-doped CdS nanowires by a simple thermal evaporation method and demonstrated their interesting optical properties. XRD and Raman scattering spectrum confirmed that Sn(IV) was doped into CdS nanowires successfully. Both near bandgap emission and trapped-state emission were observed simultaneously in doped single nanowire. Further analysis of trapped-state emission versus near bandgap emission indicates a nonlinear electron–phonon coupling. The doped CdS nanowires with variable Sn dopant concentration revealed well separated multi-phonon replica (1LO, 2LO, 4LO)-assisted emission bands, demonstrating the enhanced electron–phonon coupling and significant trapped state. The lifetime decay suggested multi-component relaxations and reflected variable recombination channels of photo-generated carriers. These Sn-doped CdS nanowires may find potential application in light emitting device and nano-photonics system.

## Data Availability

The datasets used or analyzed during the current study are available from the corresponding author on reasonable request.

## Abbreviations

PL: Photoluminescence
1D: One-dimensional
EHP: Electron–hole plasma
F-P: Fabry–Perot
SCCM: Standard cubic centimeters per minute
VLS: Vapor–liquid–solid
